# A Practical Guide to Resonance Frequency Assessment for Heart Rate Variability Biofeedback

**DOI:** 10.3389/fnins.2020.570400

**Published:** 2020-10-08

**Authors:** Fred Shaffer, Zachary M. Meehan

**Affiliations:** ^1^Center for Applied Psychophysiology, Truman State University, Kirksville, MO, United States; ^2^Department of Psychological and Brain Sciences, University of Delaware, Newark, DE, United States

**Keywords:** biofeedback, complexity, emotional self-regulation, heart rate variability, neurocardiology, resonance, performance

## Abstract

Heart rate variability (HRV) represents fluctuations in the time intervals between successive heartbeats, which are termed interbeat intervals. HRV is an emergent property of complex cardiac-brain interactions and non-linear autonomic nervous system (ANS) processes. A healthy heart is not a metronome because it exhibits complex non-linear oscillations characterized by mathematical chaos. HRV biofeedback displays both heart rate and frequently, respiration, to individuals who can then adjust their physiology to improve affective, cognitive, and cardiovascular functioning. The central premise of the HRV biofeedback resonance frequency model is that the adult cardiorespiratory system has a fixed resonance frequency. Stimulation at rates near the resonance frequency produces large-amplitude blood pressure oscillations that can increase baroreflex sensitivity over time. The authors explain the rationale for the resonance frequency model and provide detailed instructions on how to monitor and assess the resonance frequency. They caution that patterns of physiological change must be compared across several breathing rates to evaluate candidate resonance frequencies. They describe how to fine-tune the resonance frequency following an initial assessment. Furthermore, the authors critically assess the minimum epochs required to measure key HRV indices, resonance frequency test-retest reliability, and whether rhythmic skeletal muscle tension can replace slow paced breathing in resonance frequency assessment.

## Introduction

Slow paced breathing is a central component of HRV biofeedback because respiratory sinus arrhythmia (RSA) amplitude (peak-to-trough heart rate difference across the breathing cycle) increases with slow breathing (Cooke et al., 1998). The resonance frequency training model identifies the respiration rate that produces the greatest heart rate oscillations by stimulating the *baroreflex*, which is the homeostatic system that regulates blood pressure using *baroreceptors* (blood pressure receptors; Swenne, 2013). This protocol measures HRV changes as adult clients breathe from 6.5 to 4.5 breaths per min (bpm) in 0.5-bpm steps (Lehrer et al., 2003).

The purpose of this article is to describe Lehrer and colleagues’ resonance frequency assessment protocol in detail, illustrate the challenges in choosing between several potential resonance frequencies, and address issues like test-retest validity that require further research. We organized this article so that sections build on each other. These include: HRV, Baroreflex, Vaschillo Two Closed-Loop Model, Resonance Frequency Model, Importance of Resonance Frequency Assessment, Individualized Frequencies, Resonance Frequency Assessment Protocol, Resonance Frequency Selection, and Unanswered Questions.

The HRV section begins with an explanation of HRV, its relationship to HR, how breathing can produce large-scale HR oscillations called RSA, and the clinical and performance applications of resonance frequency training. The authors emphasize that HRV indexes neurocardiac function and autonomic functioning, as well as the mobilization and use of scarce self-regulatory resources. Finally, this section reviews how healthy variability contributes to regulatory capacity and adaptability.

The Baroreflex section explains the baroreflex mechanism and how breathing at slow rates (e.g., 4.5 to 6.5 bpm) produces the large-scale heart rate changes observed in RSA. The Vaschillo Two Closed-Loop Model section introduces an evidence-based model of how activities like slow paced breathing and rhythmic skeletal muscle tension can stimulate the baroreflex and increase RSA. The Resonance Frequency Model section introduces the concept of resonance, explains that the volume of blood in the vascular tree determines each individual’s resonance frequency, and emphasizes that stimulation at the resonance frequency maximizes RSA and HRV.

The Importance of Resonance Frequency Assessment section summarizes preliminary evidence of the benefits of breathing at an individual’s unique resonance frequency. The Individualized Frequencies section explains the relationship between respiration rate and *peak frequency* (largest amplitude frequency) and stresses that the respiration rate that produces a peak frequency depends on the location of the resonance frequency between 4.5 and 6.5 bpm. Resonance frequency training does not always reward 6 bpm (0.1 Hz) breathing because the resonance frequency may be lower or higher.

The Resonance Frequency Assessment Protocol section describes sensor channels and parameters monitored, terms and definitions, normative values, client orientation, and how to conduct practice breathing and resonance frequency assessment trials. The Resonance Frequency Selection section summarizes resonance frequency assessment criteria and explains the importance of *phase synchrony* (alignment of heart rate and respiration rate signal peaks) and *HR Max – HR Min* (the mean difference between the highest and lowest heart rate across all breathing cycles). This section describes procedures for breaking ties and confirming the resonance frequency during the second session with a client.

The Unanswered Questions section explores why resonance frequency assessment is necessary given that slow paced breathing increases HRV and achieves clinical gains without HRV biofeedback. We revisit evidence that training at a client’s resonance frequency may improve systolic and mood. Resonance Frequency Test-Reliability presents evidence of acceptable 2-week test-retest reliability for the Lehrer et al. (2003) resonance frequency assessment protocol and stresses the need for replication studies with more robust samples. Finally, Rhythmic Skeletal Muscle Tension summarizes evidence that this technique can also increase RSA and HRV and calls for research concerning its comparability to resonance frequency breathing and when resonance frequency assessment using this method can achieve acceptable test-retest validity.

## HRV

Heart rate, HRV, and RSA calculations depend on the time intervals between heartbeats (Task Force Report, 1996). Heart rate is the number of heartbeats each min. Along with associated metrics, it provides detailed information that clinicians can apply in a variety of medical and psychological interventions (Lehrer et al., 2020a). Frequently used as a target in clinical and performance interventions, HRV represents fluctuations in the time intervals between successive heartbeats, which are termed interbeat intervals (Task Force Report, 1996). Clinicians measure these interbeat intervals in milliseconds (ms). For example, a 60-bpm heart rate corresponds to an interbeat interval of 1000 ms since there are sixty 1000-ms intervals in a min. HRV biofeedback presents heart rate and sometimes also directly some HRV parameters to individuals to improve their affective, cognitive, and cardiovascular functioning (McCraty and Shaffer, 2015). These changes may be mediated by increased cardiac vagal tone, RSA, and activation of integrated homeostatic systems. The goal of HRV biofeedback is to increase RSA, which is heart rate acceleration and deceleration across the breathing cycle (Eckberg, 1983), in order to enhance autonomic homeostatic capacity (Vaschillo et al., 2002, p. 4). RSA involves respiration-driven changes in heart rate that are mediated by the *vagus nerve*, which conveys baroreceptor inputs to the brain and then returns them to the heart after integration in the brain (Kollai and Mizsei, 1990). When we inhale, the cardiovascular center inhibits vagal firing and heart rate speeds (Yasuma and Hayano, 2004). Conversely, when we exhale, the cardiovascular center restores vagal inhibition and heart rate slows (Eckberg and Eckberg, 1982; Berntson et al., 1997). HRV biofeedback teaches clients to increase RSA by creating sinusoidal phase-synchronous patterns of heart rate and respiration (Lehrer and Gevirtz, 2014). HRV biofeedback is extensively used to treat an array of disorders (e.g., asthma and depression) and enhance performance in a variety of contexts (e.g., sports; Gevirtz, 2013; Tan et al., 2016; Lehrer et al., 2020a). While the final targets of these applications may differ, HRV biofeedback increases cardiac vagal activity (Vaschillo et al., 2006; Lehrer et al., 2020a) and stimulates the negative feedback loops that are responsible for homeostasis (Lehrer and Eddie, 2013).

The neurovisceral integration model describes HRV as an emergent property of complex cardiac-brain interactions and non-linear autonomic nervous system (ANS) processes. HRV provides a window into neurocardiac function. HRV may reflect medial prefrontal cortex (mPFC) integration with brainstem regulation of the heart by the nucleus tractus solitarius (Thayer et al., 2012). The neurovisceral integration model describes the interrelationship between the prefrontal cortex, HRV, and executive function (Thayer et al., 2009). In turn, increased HRV may enhance top-down mPFC control of emotional health (Mather and Thayer, 2018).

Heart rate variability is generated by interdependent regulatory systems with widely varying rhythms that enable us to adapt to physical and psychological challenges (Thayer and Lane, 2000). Short-term (∼5 min) HRV measurements are produced by interactions among the autonomic, cardiovascular, central nervous, endocrine, and respiratory systems. These integrated systems utilize feedback from *baroreceptors* (receptors that detect blood pressure changes) and *chemoreceptors* (receptors that monitor chemicals like blood gases; Kougias et al., 2010).

Heart rate variability is a marker for the regulation of integrated functions and efficient allocation of limited self-regulatory resources. HRV appears to index autonomic functioning, blood pressure, cardiac functioning, digestion, oxygen and carbon dioxide exchange, vascular tone (diameter of resistance vessels), and possibly facial muscle regulation (Gevirtz et al., 2016). HRV reflects the vagal contribution to executive functions, affective control, and social self-regulation (Byrd et al., 2015; Laborde et al., 2017; Mather and Thayer, 2018). Indeed, Laborde et al. (2018) *vagal tank theory* proposes that vagal traffic to the heart indicates how efficiently we mobilize and use scarce self-regulatory resources.

Both regulatory capacity and adaptability depend on healthy variability due to increased vagal traffic. “A healthy heart is not a metronome” (Shaffer et al., 2014, p. 5). Variability enables adaptability. Multiple overlapping system oscillations, characterized by mathematical chaos, produce the complex non-linear oscillations of a healthy heart. “Oscillatory patterns with greater complexity, such as those that occur when a number of oscillatory patterns overlap, are described as ‘chaotic.’ Chaos reflects the simultaneous operation of numerous control processes” (Lehrer and Eddie, 2013, p. 145). The integrated action of multiple control systems contributes stability in response to challenges like exercise and stressors. Practically, the interdependence of these control systems means that interventions like slow paced breathing can initiate system-wide changes to increase HRV (Goldberger, 1991; Lehrer and Eddie, 2013). Slow paced breathing at the rate of 6 breaths per min produces large-scale increases in cardiorespiratory synchrony (Noble and Hochman, 2019). Healthy variability allows rapid responses to changing workloads and unpredictable environmental challenges (Beckers et al., 2006) and contributes to regulatory capacity (Grossman and Taylor, 2007). Whereas healthy biological systems show spatial and temporal complexity, diseases like cardiac conduction disorders can decrease or increase complexity (Vaillancourt and Newell, 2002). Increased HRV is only desirable when it is produced by increased cardiac vagal tone instead of cardiac conduction abnormalities (Stein et al., 2005).

## The Baroreflex

**FIGURE 1:**
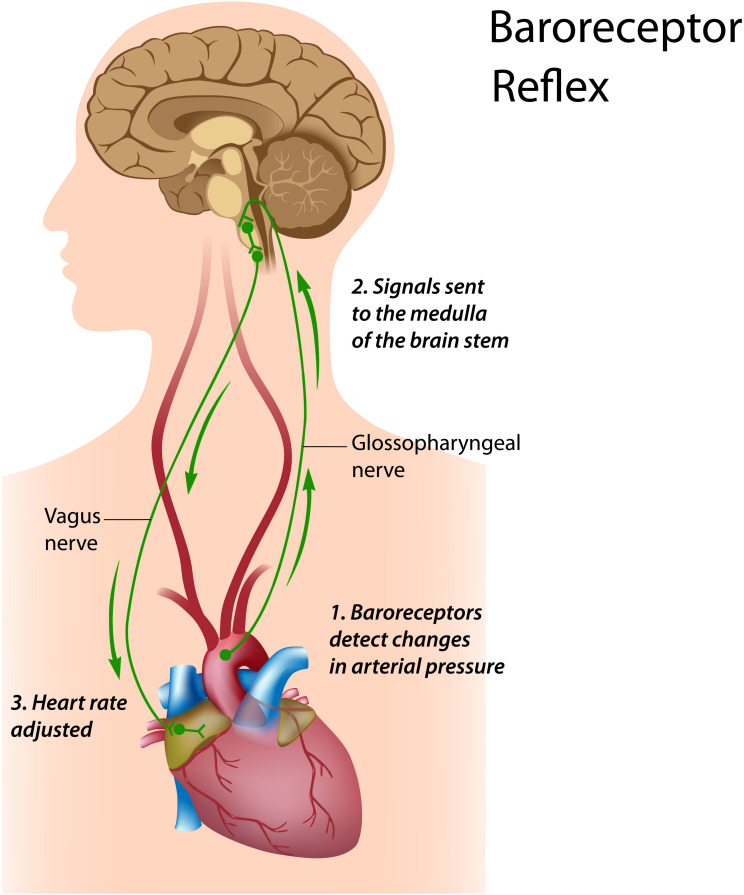
Baroreceptor reflex. Royalty-free stock photo. Credit: Alila Sao Mai/Shutterstock.com. In the baroreceptor reflex: (1) baroreceptors located in the aortic arch and internal carotid arteries detect a rise in blood pressure and increase their firing rate; (2) these signals reach the nucleus tractus solitarius in the medulla; and (3) the nucleus tractus solitarius sends signals to the sinoatrial node of the heart via the vagus nerve to slow its rate of contraction.

**FIGURE 2:**
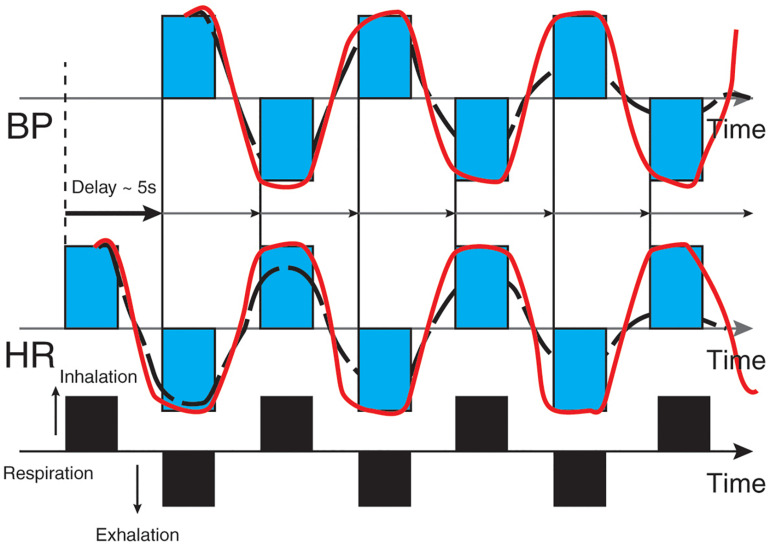
Heart rate and blood pressure oscillations elicited by respiration. Credit and permissions: adapted from Evgeny Vaschillo. Original publication: Lehrer and Vaschillo (2008). The future of HRV biofeedback. *Biofeedback* 36(1), 11–14. This graphic depicts blood pressure oscillations on the top and heart rate oscillations on the bottom. Inhalation causes an immediate rise in heart rate, followed (∼5 s) by increased blood pressure and baroreceptor firing. Exhalation results in an immediate decrease in heart rate followed (∼5 s) by decreased blood pressure and baroreceptor firing.

The *baroreflex* is central to understanding HRV biofeedback because maneuvers like slow paced breathing and rhythmic skeletal muscle tension stimulate it to increase RSA. Rhythmic skeletal muscle tension involves the simultaneous contraction of the hands and feet at rates from 6.5 to 4.5 contractions per min (cpm) while sitting. Both slow paced breathing and rhythmic skeletal muscle tension are hypothesized to increase HRV by stimulating a ∼0.1-Hz resonance in the cardiovascular system (Vaschillo et al., 2011). The baroreflex, which provides homeostatic control of acute changes, continuously operates through the interaction of multiple regulatory systems. Cardiorespiratory control of blood pressure and HRV depends on baroreceptors found in the aortic arch and carotid sinuses. While these blood pressure sensors continuously generate action potentials, blood pressure modulates their firing rate. Rising blood pressure increases and falling blood pressure decreases afferent transmission via glossopharyngeal (IX) and vagus (X) nerves that targets the nucleus tractus solitarius in the dorsomedial medulla. The nucleus tractus solitarius, in turn, directs the medulla’s vasomotor and cardiac control centers to adjust vascular tone and heart rate, respectively (Figure 1; Cutsforth-Gregory and Benarroch, 2017; Fox and Rompolski, 2019). The baroreflex integrates blood pressure, heart rate, and vascular tone control systems (Vaschillo et al., 2002) and contributes to RSA (Karemaker, 2009) along with a brainstem respiratory central pattern generator (Berntson et al., 1997; Eckberg, 2003) and pulmonary afferents (Taha et al., 1995; Koh et al., 1998). Respiration produces blood pressure and heart rate oscillations at an individual’s resonance frequency. “… as you inhale, HR rises and BP falls, but the baroreflex causes an immediate augmentation of the respiration-induced HR increase, with the opposite happening as you exhale, causing high-amplitude HR oscillations” (Lehrer and Vaschillo, 2008, p. 12; Figure 2).

## Vaschillo’s Two Closed-Loop Model

Vaschillo’s two closed-loop model explains how HRV biofeedback procedures like slow paced breathing and rhythmic skeletal muscle tension can stimulate the baroreflex and amplify RSA. Vaschillo et al. (2002) describe the vascular tone and heart rate baroreflexes as closed loops and propose that stimulating one closed loop activates its counterpart. HRV biofeedback typically utilizes *slow paced breathing*, which is breathing at a target rate such as 6 bpm, to stimulate the baroreflex and increase RSA (Gevirtz et al., 2016). Respiration can produce blood pressure oscillations via changes in thoracic pressure (Pinsky, 2018) that can stimulate the closed loops. Rhythmic skeletal muscle tension may produce comparable changes by stimulating blood pressure, heart rate, and vascular tone control systems without the requirement of slower-than-normal respiration (Vaschillo et al., 2011).

## Resonance Frequency Model

*Resonance* is an amplification process in which stimulating a negative feedback, self-corrective system at its intrinsic frequency generates high-amplitude oscillations at that frequency (Başar, 1998; Lehrer and Gevirtz, 2014). Resonance is a property of the baroreflex system (Vaschillo et al., 2002, 2006). The mechanisms responsible for this amplification are complex:

The mechanism for this effect lies in a confluence of processes: (1) phase relationships between heart rate oscillations and breathing at specific frequencies, (2) phase relationships between heart rate oscillations and breathing at specific frequencies, (3) activity of the baroreflex, and (4) resonance characteristics of the cardiovascular system (Lehrer and Gevirtz, 2014, p. 1).

The resonance frequency model predicts that we can best stimulate the baroreflex and increase RSA and HRV at an individual’s unique resonance frequency. In the cardiovascular system, the volume of blood in the vascular tree is responsible for the delay in the entire baroreflex loop across inhalation and exhalation (Vaschillo et al., 2011; Sakakibara et al., 2020). The *resonance frequency model* proposes that breathing, rhythmic skeletal muscle tension, and emotional stimulation (e.g., viewing positive and negative emotionally charged slides) at the resonance frequency (∼ 0.1 Hz) can increase RSA and HRV (Vaschillo et al., 2006). This phenomenon resembles striking a bell that continues to resonate. At the resonance frequency in adults, when heart rate rises during inhalation blood pressure starts to fall ∼ 5 s later. The strength of heart rate oscillations increases 4–10 times from resting baselines (difference between minimum and maximum heart rate) (Vaschillo et al., 2002; Lehrer et al., 2020b). External stimulation such as slow paced breathing or rhythmic skeletal muscle tension near an individual’s precise resonance frequency produces the greatest RSA and HRV and increases *baroreflex gain* (heart rate change per 1 mmHg change in blood pressure; Lehrer and Gevirtz, 2014).

## The Importance of Resonance Frequency Assessment

The purpose of assessment is to discover the baroreflex resonance frequency. While all HRV biofeedback training protocols are designed to stimulate the baroreflex, resonance frequency and 6-bpm slow paced breathing approaches may target different frequencies when adults are taller. Although several researchers train individuals to breathe at their resonance frequency (Lehrer et al., 2004; Lin et al., 2012; Steffen et al., 2017), others simply instruct them to breathe at slower-than-normal rates (Zautra et al., 2010; Cullins et al., 2013). There is compelling evidence that breathing near an individual’s resonance frequency – but not at the exact rate – increases RSA amplitude and baroreflex gain compared with other frequencies (Vaschillo et al., 2002, 2004; Lehrer et al., 2020a). However, we lack conclusive evidence that resonance frequency breathing produces superior clinical outcomes in treatment of most disorders (Lehrer et al., 2020a). Researchers have started to address this question and initial findings support the importance of training at the resonance frequency. For instance, 5 weeks of resonance frequency training produced greater systolic blood pressure reductions in prehypertensive participants than slow paced breathing (Lin et al., 2012). In a second study, 15 min of slow paced breathing at the resonance frequency produced more positive mood than resonance frequency + 1 bpm or control groups and lower systolic blood pressure than the control group during the Paced Auditory Serial Addition Task (Steffen et al., 2017).

## HRV Biofeedback Trains Clients at Individualized Frequencies

Heart rate variability biofeedback stimulates the baroreflex at a given rate to produce a peak frequency in the LF range through exercises such as slow paced breathing (Gevirtz et al., 2016). Respiration rate determines the *electrocardiogram’s* (ECG’s) peak frequency (Shaffer and Ginsberg, 2017). For example, breathing at 6 bpm produces a peak frequency of 0.1 Hz because (6 breaths/min)/(60 s/min) = 0.1 breaths/s [0.1 Hz]. We show adult peak frequencies and their corresponding respiration rates (bpm) in Table 1. Depending on where an adult’s resonance frequency lies between 4.5 and 6.5 bpm, slow paced breathing could produce a peak frequency between 0.075 and 0.11 Hz (Vaschillo et al., 2002, 2006). In practice, we provide auditory and visual feedback to reward increases in the amplitude of this frequency, which may differ from 0.1 Hz, to maximize RSA amplitude and baroreflex gain (Lehrer and Gevirtz, 2014).

**TABLE 1:** Respiration rates and corresponding ECG peak frequencies.

Respiration rate	Peak frequency (Hz)
4.5	0.075
5.0	0.08
5.5	0.09
6.0	0.10
6.5	0.11
7.0	0.12
7.5	0.13

*Credit: Center for Applied Psychophysiology. Respiration rate, breaths per min; peak frequency, highest amplitude electrocardiogram frequency.*

## A Resonance Frequency Assessment Protocol

Resonance frequency assessment identifies the unique breathing rate that best stimulates the baroreflex and maximizes RSA amplitude before initiating HRV biofeedback. The resonance frequency ranges from 4.5 to 6.5 bpm for adults and 6.5 to 9.5 bpm for children. The difference by age group arises because children are typically smaller than adults, and therefore, have smaller vascular trees and less inertia due to blood volume (Lehrer and Gevirtz, 2014). Resonance frequency measurement (Lehrer et al., 2013) has greatly influenced HRV biofeedback practice and can be readily adapted for different age groups and morbidities. The main adjustments for children include simplified instructions, providing entertaining displays to engage them, and running their slow paced breathing trials from 9.5 to 6.5 bpm instead of the adult range from 6.5 to 4.5 bpm. No special procedural adjustments are required for healthy older adults. They tend to have lower resting RSA and smaller RSA increases following HRV biofeedback (Lehrer et al., 2020a). Adult complaints like asthma, Generalized Anxiety Disorder, and low back pain, as well as dysfunctional breathing behaviors like overbreathing may be associated with rapid breathing. Postpone resonance frequency assessment until you successfully train them to breathe effortlessly (Peper et al., 2008) between 4.5 and 6.5 bpm. Because slower breathing may be difficult for some clients, they may overbreathe and expel excessive CO_2_. If they report that they feel faint or that their heart is beating too hard, instruct them to take shallower and smoother breaths (Lehrer et al., 2013). Resonance frequency assessment is contraindicated for clients whose sinus rhythm is driven by a pacemaker because this device externally regulates HRV. Assessment may also be contraindicated for clients whose overbreathing compensates for increased acidity in the blood due to conditions like kidney disease. Slow paced breathing would increase CO_2_ levels in the blood and dangerously increase acidosis (Khazan, 2013).

### Sensor Channels and Parameters Monitored

In practice, the resonance frequency measurement protocol requires the ability to display instantaneous HR and respiration in real time. A clinician monitors HR using an ECG (or a *photoplethysmograph* (PPG), which optically measures the maximum value of the pulse wave to calculate instantaneous heart rate and interbeat intervals. Both ECG and PPG sensors obtain equivalent interbeat interval values under resting conditions with normal tissue perfusion (Giardino et al., 2002; Schafer and Vagedes, 2013). During slow paced breathing, PPG monitoring from the toes (2.1 beats), thumb (2.9 beats), and earlobes (3.4 beats) produces increasing phase delay with respect to the ECG. There is bilateral symmetry in phase delay between homologous recording sites (Allen, 2019). During resting conditions following 1 min of deep breathing, PPG recordings from the ear lobe achieve good agreement with ECG measurements (Weinschenk et al., 2016); however, the PPG method may measure HRV less accurately during slow paced breathing (Jan et al., 2019). In addition, ECG values are more accurate than PPG values when there is marked sympathetic activation – typically occurring in disorders such as anxiety – as peripheral vasoconstriction affects detection of the peak of the blood pressure wave from the digits but not R-spike detection from the chest, torso, or wrists (Giardino et al., 2002; Schafer and Vagedes, 2013; Shaffer and Combatalade, 2013). To determine when the ECG method is more appropriate, a clinician should evaluate the raw PPG waveform before data collection to decide whether it is flat or low amplitude (Shaffer and Combatalade, 2013).

Respiratory feedback serves several functions during resonance frequency assessment as adults breathe from 6.5 to 4.5 bpm in 0.5-bpm steps: pacing, respiration rate confirmation, and identification of dysfunctional breathing. A respiration display guides clients to breathe at prescribed rates and confirms their success. Both actions are critical because we cannot evaluate the effects of breathing at 5.5 bpm if a client actually breathed at 6 bpm. Respiratory monitoring is also essential to identify dysfunctional breathing behaviors like *apnea* (breath-holding) and *overbreathing* (excessive exhaling of CO_2_), which can interfere with HRV biofeedback. Although HRV analysis software (e.g., Kubios) can extract respiration data *after* a slow paced breathing trial, resonance frequency assessment using slow paced breathing requires the real-time display of respiration to ensure that clients breathe at precise rates. A better solution than extracting respiration data after each slow paced breathing trial is to monitor breathing using a *respirometer* (flexible sensor band) that measures abdominal or thoracic expansion and contraction to acquire the respiratory waveform (Shaffer and Moss, 2019). A clinician should continuously monitor all raw waveforms (ECG or PPG, and respirometer) for artifact (false values) during each slow paced breathing trial so that they can immediately repeat contaminated trials.

### Terms and Definitions

Data from heart rate, respiration, and their synchrony provide detailed information for resonance frequency assessment. Clinicians who assess resonance frequency are concerned with the smoothness and regularity of heart rate signals. *Heart rate-respiration phase synchrony* is the phase angle of the peaks and troughs of the heart rate and respiration rate signals. *HRV frequency-domain metrics* calculate the spectral distribution of signal energy. HRV biofeedback is concerned with the *very-low-frequency* (VLF; 0.0033–0.04 Hz), *low-frequency* (LF; 0.04–0.15 Hz), and *high-frequency* (HF; 0.15–0.40 Hz) bands. Although there is uncertainty regarding the sources of VLF power in short-term measurements (Kleiger et al., 2005), sympathetic activation due to effortful breathing is a possible source (Bernardi et al., 1996). There is also disagreement about the sources of LF power in short-term measurements (Akselrod et al., 1981; Goldstein et al., 2011; Reyes del Paso et al., 2013). LF power is an important indicator of HRV biofeedback training success for several reasons. First, slow paced breathing, which is one method of stimulating the baroreflex, increases LF power by increasing cardiac vagal tone (Kromenacker et al., 2018). Second, increased LF power is associated with greater RSA and HRV (Vaschillo et al., 2002). Increased RSA and HRV occur in the LF range because the baroreceptor reflex’s resonance frequency resides within this range (Lehrer et al., 2020a). HF power is due to parasympathetic activity, and the natural logarithm of HF power indexes cardiac vagal tone (Task Force Report, 1996). *Absolute power* is the signal energy within a frequency band expressed in ms^2^/Hz. *Normalized power* is the percentage of total power. For example, normalized LF power is LF/(LF + HF) or LF/(VLF + LF + HF). *Peaks* are the highest-amplitude frequencies within a band like LF. Resonance frequency assessment examines both the magnitude and number of LF peaks (Shaffer and Ginsberg, 2017). LF peak amplitude and the number of LF peaks are among six resonance frequency selection criteria (Lehrer et al., 2013). Larger peaks indicate greater resonance effects due to increased breathing and heart rate synchrony. Several LF peaks may occur when individuals breathe at a single rate outside of their resonance frequency. This can produce separate peaks at the baroreflex frequency and their actual respiratory rate. While breathing at adjacent rates may still stimulate the baroreflex, it will produce smaller resonance effects (Lehrer et al., 2020a). When clients do not precisely follow instructions, changing respiration rates can also produce multiple peaks and weaker resonance effects. Breathing in a narrow frequency range around the resonance frequency better stimulates the baroreflex and increases RSA than breathing in a wider frequency range (Vaschillo et al., 2002).

*HRV time-domain indices* quantify the amount of variability in a series of interbeat intervals. For example, *HR Max-HR Min* is the average change between the highest and lowest heart rate across all breathing cycles (Cipresso et al., 2019).

During resonance frequency assessment, clinicians can measure respiration rate, heart rate, heart rate-respiration phase synchrony, heart rate peak-trough amplitude, mean LF power, the magnitude and number of peaks within the LF band, and the smoothness of the heart rate curve envelope during each breathing trial. These data will allow clinicians to compare the differential effects of breathing rates on HRV parameters to identify each client’s resonance frequency (Lehrer et al., 2013). Where clinical or peak performance interventions require more comprehensive information, clinicians can integrate a sphygmomanometer, capnometer, electrodermograph, and electromyograph (EMG) into psychophysiological assessment. A *sphygmomanometer* measures systolic and diastolic blood pressure. A *capnometer*, which monitors *end-tidal* CO_2_ (alveolar CO_2_ concentration at the conclusion of a breath), can detect overbreathing. This dysfunctional breathing behavior involves excessive CO_2_ exhalation due to mouth breathing, rapid deep breathing, and sighs, and yawns (Khazan, 2013, 2019a). An *electrodermograph*, which measures eccrine sweat gland activity, can disclose increased sympathetic nervous system arousal that may accompany dysfunctional breathing. *Skin conductance level* (SCL) is a tonic measure of eccrine sweat gland activity. Furthermore, an *EMG*, which monitors skeletal muscle action potentials, can likewise indicate dysfunctional breathing if frontales or breathing accessory muscles exceed normal resting values of ≤3 microvolts (μV; Shaffer, 2020). For example, elevated frontales, scalene, or trapezius EMG activity may signal excessive breathing effort (Khazan, 2013).

The decision to add these modalities involves a cost/benefit analysis. Is the extra information worth the cost in equipment and time? Clinicians might answer this question on a case-by-case basis, guided by the client’s training goals and whether the intervention is 6-bpm slow paced breathing or resonance frequency biofeedback. For example, if the training goal is to lower systolic blood pressure, a sphygmomanometer can show whether one breathing rate produces greater reductions than an adjacent rate. When treating panic disorder, clinicians can use a capnometer and electrodermograph to determine which rate produces optimal end-tidal CO2 and reductions in sympathetic activation, respectively. Finally, clinicians can monitor breathing accessory muscles (trapezius and scalene) to detect overuse, as this issue may need correction regardless of the breathing rate chosen. In all these examples, clinicians should interpret patterns of psychophysiological change using adult normative values (Khazan, 2019b). Normative values obtained during *resting conditions* – no breathing instructions, feedback, or task – enable clinicians to interpret patterns of psychophysiological change during resonance frequency assessment. Because HRV time domain and frequency domain norms are influenced by age, sex, and fitness (Koenig and Thayer, 2016; Shaffer and Ginsberg, 2017), we encourage readers to consult several studies that provide representative values (Umetani et al., 1998; Berkoff et al., 2007; Nunan et al., 2010). Blood pressure should be less than 120/80 mmHg (Fox and Rompolski, 2019). End-tidal CO_2_ should range between 35 and 45 mmHg or torr. SCL should be ≤5 microsiemens (μS). Finally, EMG activity should be ≤3 μV with a wide bandpass (e.g., 20–1000 Hz; Khazan, 2019b).

### Orientation for Resonance Frequency Assessment

The resonance frequency assessment protocol (Lehrer et al., 2013) described in this article is simple to administer and provides intuitive directions:

Today, I am going to introduce you to a method that will help you control your symptoms. We will be using a number of measuring devices, and wearing them may feel a little strange in the beginning. This introduction will allow you to become familiar with what it feels like to wear the sensors, and to watch the body signals they are measuring on the screen, before we start your biofeedback training. I will attach all of the sensors to your body and then you will see what they are measuring on your monitor. These sensors will simply be measuring your physiological activity and will not cause any harm to you. I will briefly explain what each measurement is (p. 98). Attach and test each sensor, start displaying physiological activity, and explain the meaning of the graphs and numerical values. For example: In this top graph, the red line is your heart rate in terms of beats per minute, and the blue line shows your breathing. You’ll notice that the blue line moves up as you breathe in and down as you breathe out (p. 99).

Before you start resonance frequency assessment trials, invite questions and then provide a brief overview of the assessment process. As before, we encourage you to modify Lehrer et al. (2013) explanation:

Today we are going to find out the speed of breathing that should best help you to cope with your symptoms. This breathing frequency is different for each person. When you breathe at this rate, your breathing will produce strong effects on your nervous and cardiovascular systems that should be very good for you and should help you to control your symptoms (p. 99). Your heart rate varies with each breath, and with various other processes in your body, including the baroreflex. This variability is good and is a sign of health. We will now find your “resonance frequency” – the speed of breathing at which your HRV is the highest. In this task, we will ask you to breathe at five rates for periods of about 2 min each. You should not find this task difficult. However, if you feel uncomfortable at any time, you can simply stop the task and tell us. When we begin, we will ask you to breathe in and out at a 10-s breathing rate. Then we will ask you to breathe at various other rates, so we can find the exact frequency at which your cardiovascular system resonates. This will be your own resonance breathing frequency. You will be able to use this breathing rate to best help your symptoms. Breathe easily and comfortably, but not too deeply. Do not try too hard. Do you have any questions? (p. 99).

### Practice Breathing Before Resonance Frequency Trials

Clinicians should provide their clients with breathing practice before conducting resonance frequency trials because the protocol requires breathing rates that are slower than normal, especially for clinical populations such as clients diagnosed with chronic pain. Although a healthy resting adult breathes from 12 to 20 bpm (Khazan, 2019a), a resonance frequency assessment protocol instructs adults to breathe at less than half that rate. Allow clients to practice relaxed breathing from 5.5 to 6 bpm before starting resonance frequency trials. When a respiration rate is difficult, instruct them to increase or decrease it by 1/2 bpm. For example, if a client typically breathes at 18 bpm, instruct them to decrease their breathing rate every few seconds from 18 bpm to 17.5 bpm to 17 bpm, and so forth. Clinicians should standardize the inhalation-to-exhalation ratio across breathing trials. Longer exhalation than inhalation is recommended in resonance frequency assessment (Lehrer et al., 2013) and may increase RSA from baseline values due to a greater increase in cardiac vagal tone (Strauss-Blasche et al., 2000; Van Diest et al., 2014). However, several studies (Zerr et al., 2015; Meehan et al., 2017) found no difference between resting HRV metrics (e.g., HR Max-HR Min, pNN50, RMSSD, SDNN, and LF power) when participants breathed at 1:1 and 1:2 inhalation-to-exhalation ratios.

### Resonance Frequency Trials

**FIGURE 3:**
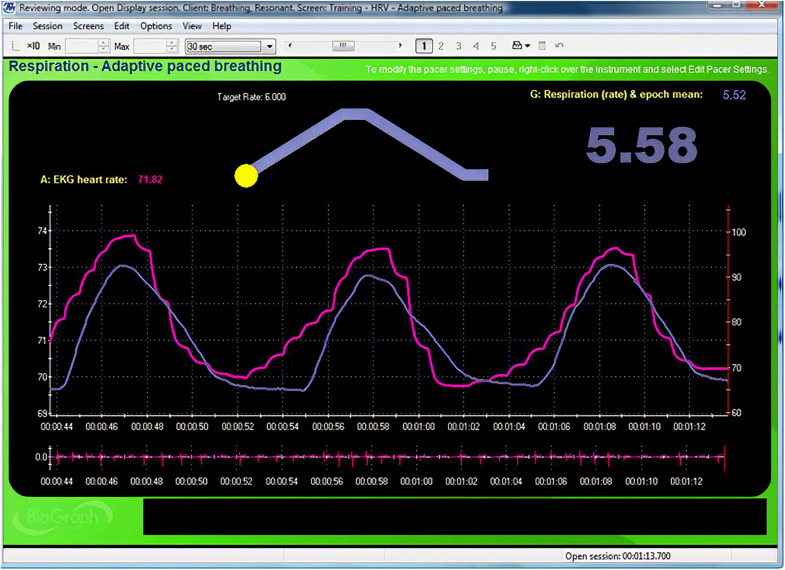
Animated pacing display. Credit and permissions: Center for Applied Psychophysiology. The top display with the moving yellow ball is designed to help clients breathe at 6 bpm. The exhalation is followed by a post-expiratory pause. The current respiration rate (5.58 bpm) appears on the right. The graph immediately below shows instantaneous heart rate (pink) and respiration (purple). Note the degree to which the waveform peaks and troughs coincide since this graphically represents phase synchrony. A raw ECG waveform is displayed toward the bottom of the screen.

Instruct your client to breathe for 2-min intervals from 6.5 to 4.5 bpm, decreasing in 0.5 bpm-steps with 2-min rest periods. Record physiological activity during slow paced breathing as separate 2-min epochs. Create a display for each resonance frequency trial and capture 2 min of raw breathing and heart rate waveforms for each respiration rate (Figure 3). Record each trial’s measurement parameters as shown in Table 2 (Lehrer et al., 2013). Valid resonance frequency assessment requires careful artifact removal because one invalid interbeat interval can significantly distort metrics like HR Max-HR Min and SDNN (Berntson et al., 1997). Although automatic artifacting can identify suspect interbeat intervals, manual artifacting may produce superior results. Please review excellent discussions of interbeat interval editing for manual artifacting (Peltola, 2012; Laborde et al., 2017), as an explanation of these strategies is outside of the scope of this article.

**TABLE 2:** Resonance frequency assessment check-list for each trial.

Resonance frequency trial parameters
pacing target bpm
actual bpm
respiration-HR phase
HR Max-HR Min
absolute LF power
normalized LF power
highest amplitude LF peak
number of distinct LF peaks
sinusoidal waveform
client difficulty

*Credit: Center for Applied Psychophysiology. bpm, breaths per min; LF, low frequency; peak, highest amplitude frequency.*

Consider the following directions when introducing each respiration rate: “Now try breathing at this frequency (following the pacer)” (Lehrer et al., 2013 p. 101). After your client completes 2 min of paced breathing, check on their comfort and verify that they followed the pacer by confirming the average respiration rate for that trial. Repeat trials if the clients were 0.25 bpm too fast or slow. *Resonance frequency assessment without a respirometer lacks this quality control; in such cases, we cannot verify that clients have breathed at the target rates*.

Check for artifactual interbeat intervals and repeat invalid epochs after the client has rested for 2 min. Examine the segment spectral display for the location of LF peaks. When a peak occurs at 4.5 or 6.5 bpm, extend assessment with trials 0.5 bpm above and below this inflection point until LF amplitude decreases.

## Resonance Frequency Selection

The goal of resonance frequency selection is to identify the frequency that best stimulates the baroreflex system and thereby increases RSA. Clinicians use six weighted criteria to evaluate adult breathing rates between 4.5 and 6.5 bpm. They prioritize these criteria by their association with resonance effects. This selection process requires careful analysis because a single breathing rate may not maximize all six criteria. When this happens, clinicians select the frequency that satisfies the majority of these criteria. The resonance frequency estimate represents the “best convergence” of the selection criteria (Lehrer et al., 2013, p. 102). Researchers have not validated these weights and they require experimental confirmation:

(1) Phase synchrony. In adults, when respiration and heart rate signals rise and fall at the same time (0°), this maximally stimulates the baroreflex and increases RSA (Vaschillo et al., 2004; Lehrer and Gevirtz, 2014; Lehrer et al., 2020a). Whether RSA optimizes pulmonary gas exchange efficiency is currently unclear (Buchheit, 2010). Software measures the phase synchrony between the respirometer and heart rate waveforms: 0° means that heart rate begins to rise at the start of an inhalation; 90° means that heart rate begins to increase during the middle of an inhalation and to decrease during the middle of an exhalation; 180° means that heart rate decreases during inhalation and increases during exhalation. Phase synchrony (∼0°) carries the greatest weight because it enables clients to achieve the greatest resonance effects. Strong resonance effects, in turn, increase RSA and many HRV metrics, and allow HRV biofeedback training to more effectively stimulate and strengthen the baroreflex (Lehrer et al., 2003, 2013).
(2) Peak-trough amplitude. Higher heart rate peak-trough amplitudes are better because greater RSA can increase baroreflex sensitivity over weeks of HRV resonance frequency training (Lehrer et al., 2003; Lehrer and Gevirtz, 2014). *HR Max – HR Min* is one method of quantifying peak-trough amplitude (Cipresso et al., 2019). Clinicians measure HR Max – HR Min using a respirometer to determine when each breathing cycle starts and ends. Peak-trough amplitude is second because larger peak-trough differences signal greater resonance effects and contribute to more effective baroreflex activation (Vaschillo et al., 2002).
(3) LF power. The baroreflex system exhibits resonance because it is a feedback system with a constant delay (Lehrer, 2013). Its resonance frequency lies within the LF range. Higher absolute and percent total LF power are desirable because they increase as the respiration rate approaches the resonance frequency and more effectively stimulates the baroreflex (Vaschillo et al., 2002). Further, cardiac vagal activity increases when individuals engage in slow paced breathing within the LF range (Kromenacker et al., 2018). Clinicians measure absolute LF power of the 0.04–0.15 Hz range in ms^2^/Hz. They calculate percent total LF power as LF/(LF + HF) or LF/(VLF + LF + HF; Lehrer et al., 2013). LF power is third because it confirms that clients are breathing at rates between 4.5 and 6.5 bpm, which are necessary to produce the greatest resonance effects and possibly RSA as well.
(4) Maximum LF amplitude peak. Larger LF peaks reflect stronger resonance effects due to greater synchrony between breathing and heart rate. Clinicians use spectral analysis to identify the LF peak with the largest absolute power (ms^2^/Hz). Maximum LF amplitude peak is fourth because the LF spectral peak is higher at the resonance frequency than at any other respiratory frequency (Lehrer et al., 2013). When clients breathe at a consistent rate within the LF range, this increases resonance effects and RSA.
(5) Smoothness of the heart rate curve envelope. Smooth heart rate waveforms are best because they permit closer phase synchrony with respiration waveforms and therefore allows clients to achieve the greatest resonance effects and RSA (Lehrer and Gevirtz, 2014). Clinicians visually inspect heart rate curve envelope for their smoothness. Signals that resemble sine waves are smooth, whereas jagged waveforms are irregular (Lehrer et al., 2013). Smoothness of the heart rate curve envelope is fifth because it reflects the breathing mechanics required to achieve the greatest resonance effects and RSA.
(6) Fewest LF peaks. Fewer peaks are better than more peaks because they are generated by breathing within a narrower frequency band about the resonance frequency within the LF range. Clients can generate multiple peaks when they breathe slightly faster or slower than their resonance frequency. This can result in a peak at the respiratory rate and another at the baroreflex frequency. In contrast to breathing at various frequencies in the LF range, breathing at a single frequency better enables phase synchrony between breathing and heart rate, stimulates the baroreflex, and increases RSA (Vaschillo et al., 2002). Clinicians can count the number of LF peaks by visually inspecting a spectral display of the LF range. The fewest LF peaks is sixth because this demonstrates that the client is *consistently* breathing within a narrow band within the LF range, which increases resonance effects and RSA.

Although it would be ideal if one respiration rate produced the greatest increases in phase synchrony, peak-trough amplitude, LF power, maximum LF amplitude peak, and heart rate curve smoothness, and the fewest LF peaks, it is unlikely. Adjacent respiration rates may optimize different selection criteria. The six criteria provide a strategy for identifying potential resonance frequencies. There were two candidate resonance frequencies in Table 3: 5.0 bpm for 53-bpm HR Max-HR Min and 5.5 bpm for 7°phase synchrony (Shaffer, 2020).

**TABLE 3:** Resonance frequency assessment of a healthy undergraduate.

Respiration rate	Phase synchrony (°)	HR Max-HR Min (bpm)	Normalized LF power (%)	Number of LF peaks	SCL (μS)	Systolic blood pressure	Diastolic blood pressure
7.5	25	40	83	+	14	103	61
7.0	22	38	94	−	14	118	65
6.5	27	43	93	−	15	133	56
6.0	13	46	95	+	15	106	73
5.5	7	49	90	+	16	116	70
5.0	−30	53	94	−	19	107	58
4.5	−32	51	94	−	20	101	72

*Credit: Center for Applied Psychophysiology. Respiration rate, breaths per min; phase synchrony, phase relationship between the peaks and troughs of heart rate and respirometer signals; HR Max-HR Min, the mean difference between the fastest and slowest heart rates across each respiratory cycle; normalized LF power, division of LF power by the sum of LF and HF power; the number of LF peaks, the number of highest amplitude frequencies within the LF range where + means fewest peaks; SCL, skin conductance level; systolic blood pressure, maximum arterial pressure during left ventricle contraction; diastolic blood pressure, minimum arterial pressure during ventricular relaxation.*

Cardiac vagal tone should be one of the resonance frequency assessment criteria since increasing this parameter is one of the goals of HRV biofeedback training (Vaschillo et al., 2006). There is evidence that both LF power and RMSSD index cardiac vagal tone when breathing at slow rates. Slow paced breathing increases LF power by increasing cardiac vagal tone (Kromenacker et al., 2018) and RMSSD reflects cardiac vagal firing with minimal confounding by respiration rate (Penttilä et al., 2001).

### How to Break Ties and Confirm the Resonance Frequency

Consider your clients’ perspective when breaking ties between nearby breathing rates and then reconfirm that rate during the first training session. Which rate feels most comfortable? If your clients struggle with breathing at 4.5 bpm, this pace may result in overbreathing and *vagal withdrawal*, in which increased sympathetic firing inhibits parasympathetic regulation (Porges, 1995; Thayer et al., 2012). Which rate brings your clients closest to their training goal? If your clients entered training to lower blood pressure, consider the rate that produces the greatest decreases. In response to the previous example, breathing at 5.0 bpm reduced blood pressure by 9/12 mmHg compared with 5.5 bpm (Shaffer, 2020). When you collaborate with your clients to break ties, you can strengthen your relationship and increase the likelihood that they will practice resonance frequency breathing outside of the clinic. After preliminary resonance frequency measurement, clinicians should monitor 3–5 min of breathing at the resonance frequency while watching for signs of overbreathing like faintness (Lehrer et al., 2013). If such symptoms are present, clinicians should encourage shallower breathing to reduce the CO_2_ loss that is responsible for them. Next, they should ask clients to breathe at rates that are 1/2-bpm faster and slower for 3–5 min each. This step allows clients to compare their subjective comfort one more time during each breathing rate. Finally, after artifacting, clinicians should evaluate the three trials – resonance frequency, resonance frequency + 1/2-bpm, and resonance frequency − 1/2-bpm – using the previous resonance frequency criteria.

## Unanswered Questions

Four major questions regarding resonance frequency assessment require further research. These questions include whether resonance frequency training is more effective than 6-bpm slow paced breathing, the minimum epoch required for valid resonance frequency measurements, the Lehrer protocol’s test-retest reliability, and whether rhythmic skeletal muscle tension can replace slow paced breathing in resonance frequency assessment.

*Does resonance frequency training produce superior outcomes in adults compared with 6-bpm slow paced breathing?* This question is an “elephant in the room” that researchers need to more completely address. While initial studies (Lin et al., 2012; Steffen et al., 2017) found evidence that resonance frequency training produces greater systolic blood pressure reductions and positive mood, a recent meta-analysis (Lehrer et al., 2020a) found non-significant effects on diastolic or systolic blood pressure.

*What minimum epoch is required to obtain valid resonance frequency measurements?* Although clinicians may assume that 2-min recordings achieve acceptable concurrent validity with respect to 5-min recordings, no peer-reviewed study has demonstrated this result for the most important resonance frequency criteria: heart rate-respiration phase synchrony and HR Max-HR Min. Different epoch lengths may be required for acceptable concurrent validity of related HRV metrics like LF and normalized LF power. Shaffer et al. (2019) evaluated the concurrent validity of these indices in 38 healthy undergraduates. Their concurrent validity criteria included a Pearson’s correlation value ≥0.90 and a Bland–Altman limits of agreement allowable difference of ± 5% of the 5-min value range. Whereas 90-s epochs were sufficient to measure LF power, 180-s records were needed to estimate 5-min normalized LF power. Researchers should investigate the concurrent validity of all four measures with a larger, more representative sample.

*How reliable is resonance frequency assessment?* Evidence of resonance frequency test-retest reliability is severely limited but encouraging. Fuller et al. (2011) reported that the resonance frequency was stable in 21 undergraduates. The authors demonstrated acceptable test-retest reliability (*r* = 0.73. *d* = 2.14) for participants assessed 2 weeks apart. The question of test-retest reliability is pivotal for resonance frequency assessment. *Why invest an entire session to measure the resonance frequency if it significantly changes across training sessions?* Researchers should replicate this finding with a larger and more representative sample. Resonance frequency assessment may achieve greater test-retest reliability in taller, rather than shorter individuals. Taller adults tend to have lower resonance frequencies due to the greater time required for blood pressure adjustment following baroreflex-mediated heart rate changes (Vaschillo et al., 2006).

*Could rhythmic skeletal muscle tension replace slow paced breathing in resonance frequency assessment?* Rhythmic skeletal muscle tension can stimulate the baroreflex like resonance frequency breathing and increase LF HRV power (Vaschillo et al., 2011). In this study, participants placed in a semi-recumbent position rhythmically contracted their hands and feet 3, 6, and 12 times per min. The rhythmic skeletal muscle tension only produced high-amplitude oscillations in blood pressure, heart rate, and vascular tone at 6 contractions per min (cpm) – which is a frequency of 0.1 Hz. These findings raise the possibility that clinicians could use rhythmic skeletal muscle tension in place of slow paced breathing to measure resonance frequency and deliver HRV biofeedback training. The rhythmic skeletal muscle tension protocol would avoid the challenging requirement that individuals breathe at unusually slow rates (e.g., 4.5–6.5 bpm). Before this protocol can be adopted, research will have to prove that it achieves acceptable criterion validity – confirmation that test scores accurately estimate scores of validated measures (Gulliksen, 1987) – with respect to slow paced breathing and test-retest validity.

## Conclusion

Variability in the timing of interbeat intervals may promote adaptive capacity. Cardiovascular health and optimal affective, cognitive, and social functioning depend on complex non-linear oscillations produced by complex neurocardiac interactions and non-linear ANS processes (Segerstrom and Ness, 2007). ANS and cardiorespiratory system plasticity make HRV biofeedback possible. The premises of the resonance frequency model of HRV biofeedback are that younger adults have a unique resonance frequency determined by the volume of blood in the vascular tree (and its inertia), and heart rate and blood pressure are 180° out of phase in younger adults at that frequency, which lies between 4.5 and 6.5 bpm or cpm. Stimulation of the baroreflex by breathing and rhythmic skeletal muscle tension near the resonance frequency can produce immediate large-scale increases in RSA compared with that at resting baselines. Weeks of HRV biofeedback training at the resonance frequency can increase baroreflex gain and cardiac vagal tone to treat clinical disorders and promote optimal performance (Lehrer et al., 2020a).

Determination of the resonance frequency is a prerequisite for HRV biofeedback resonance frequency training because adult peak frequencies range between 0.075 and 0.11 Hz. Because there is evidence that 6-bpm slow paced breathing maximizes RSA and baroreflex sensitivity (Russo et al., 2017; Zaccaro et al., 2018), is individualized training near the resonance frequency worth the expense of resonance frequency assessment and psychophysiological monitoring equipment? To date, there is preliminary evidence (Lin et al., 2012; Steffen et al., 2017) that HRV biofeedback resonance frequency training produces greater systolic blood pressure reductions than 6-bpm slow paced breathing, resonance frequency + 1 HRV biofeedback, or control conditions. Further research is needed to demonstrate the value of resonance frequency assessment and training.

The resonance frequency protocol described in this article can be readily adapted for different ages and morbidities. Resonance frequency assessment is contraindicated in medical conditions that produce acidosis and where a pacemaker externally controls the sinus rhythm. This protocol requires monitoring of the heart rate and respirometer waveforms to measure heart rate-respiration phase synchrony and HR Max-HR Min, which are its most important criteria. Although both ECG and PPG methods produce comparable results when clients breathe at normal rates with healthy tissue perfusion, the ECG is more accurate during slow paced breathing and when sympathetic activation results in vasoconstriction and smaller pulse wave peaks. The resonance frequency should meet most of Lehrer et al. (2013) weighted criteria after data have been carefully artifacted. Clinicians should incorporate both LF power and RMSSD as selection criteria since they index cardiac vagal tone. Because several breathing rates may maximize different resonance frequency criteria, clinicians may break ties by considering client comfort, preference, and training goals.

We have raised several important questions. Does resonance frequency training produce superior outcomes in adults compared with 6-bpm slow paced breathing? Are 2 min sufficient to measure the HRV indices used to determine the resonance frequency? What is the 2-week test-retest reliability for the resonance frequency? Can rhythmic skeletal muscle tension replace paced breathing in resonance frequency assessment? Answers to these questions could refine Lehrer and colleagues’ assessment protocol and increase confidence in its results.

## Ethics Statement

Written informed consent was obtained from the individual(s), and/or minor(s)’ legal guardian/next of kin, for the publication of any potentially identifiable images or data included in this article.

## Author Contributions

FS reviewed the literature, wrote the initial manuscript, and made subsequent revisions following feedback from ZM. ZM reviewed the literature, created and maintained a UST publication database, and made editorial suggestions for all drafts. FS and ZM discussed the resonance frequency assessment literature and developed the central themes of this review manuscript. Both authors contributed to the article and approved the submitted version.

## Conflict of Interest

The authors declare that the research was conducted in the absence of any commercial or financial relationships that could be construed as a potential conflict of interest.

## References

[B1] Akselrod S. Gordon D. Ubel F. A. Shannon D. C. Barger A. C. Cohen R. J. (1981). Power spectrum analysis of heart rate fluctuation: aquantitative probe of beat-to-beat cardiovascular control. *Science* 213 220–222. 10.1126/science.6166045 6166045

[B2] Allen J. (2019). Quantifying the delays between multi-site photoplethysmography pulse and electrocardiogram R-R interval changes under slow-paced breathing. *Front. Physiol.* 10:1190. 10.3389/fphys.2019.01190 31607946 PMC6774289

[B3] Başar E. (1998). “Resonance phenomena in the brain, physical systems, and nature,” in *Brain Functions and Oscillations*, ed. Başar E. (Berlin: Springer Verlag). 10.1007/978-3-642-72192-2

[B4] Beckers F. Verheyden B. Aubert A. E. (2006). Aging and nonlinear heart rate control in a healthy population. *Am. J. Physiol. Heart Circ. Physiol.* 290 H2560–H2570. 10.1152/ajpheart.00903.2005 16373585

[B5] Berkoff D. J. Cairns C. B. Sanchez L. D. Moorman C. T. (2007). Heart rate variability in elite American track-and-field athletes. *J. Strength Cond. Res.* 21 227–231. 10.1519/00124278-200702000-00041 17313294

[B6] Bernardi L. Valle F. Coco M. Calciati A. Sleight P. (1996). Physical activity influences heart rate variability and very-low-frequency components in Holter electrocardiograms. *Cardiovasc. Res.* 32 234–237. 10.1016/0008-6363(96)00081-8 8796109

[B7] Berntson G. G. Bigger J. T. Jr. Eckberg D. L. Grossman P. Kaufmann P. G. Malik M. (1997). Heart rate variability: origins, methods, and interpretive caveats. *Psychophysiology* 34 623–648. 10.1111/j.1469-8986.1997.tb02140.x 9401419

[B8] Buchheit M. (2010). Respiratory sinus arrhythmia and pulmonary gas exchange efficiency: time for a reappraisal. *Exp. Physiol.* 95:767. 10.1113/expphysiol.2010.053470 20554926

[B9] Byrd D. L. Reuther E. T. McNamara J. P. H. DeLucca T. L. Berg W. K. (2015). Age differences in high frequency phasic heart rate variability and performance response to increased executive function load in three executive function tasks. *Front. Psychol.* 5:1470. 10.3389/fpsyg.2014.01470 25798113 PMC4350398

[B10] Cipresso P. Colombo D. Riva G. (2019). Computational psychometrics using psychophysiological measures for the assessment of acute mental stress. *Sensors* 19:781. 10.3390/s19040781 30769812 PMC6412878

[B11] Cooke W. H. Cox J. F. Diedrich A. M. Taylor J. A. Beightol L. A. Ames J. E. (1998). Controlled breathing protocols probe human autonomic cardiovascular rhythms. *Am. J. Physiol.* 274 H709–H718. 10.1152/ajpheart.1998.274.2.H709 9486278

[B12] Cullins S. W. Gevirtz R. N. Poeltler D. M. Cousins L. M. Harpin R. Muench F. (2013). An exploratory analysis of the utility of adding cardiorespiratory biofeedback in the standard care of pregnancy-induced hypertension. *Appl. Psychophysiol. Biofeedback* 38 161–170. 10.1007/s10484-013-9219-4 23613006

[B13] Cutsforth-Gregory J. K. Benarroch E. E. (2017). Nucleus of the solitary tract, medullary reflexes, and clinical implications. *Neurology* 88 1187–1196. 10.1212/WNL.0000000000003751 28202704

[B14] Eckberg D. L. (1983). Human sinus arrhythmia as an index of vagal cardiac outflow. *J. Appl. Physiol. Respir. Environ. Exerc. Physiol.* 54 961–966. 10.1152/jappl.1983.54.4.961 6853303

[B15] Eckberg D. L. (2003). The human respiratory gate. *J. Physiol.* 548 339–352. 10.1113/jphysiol.2003.037192 12626671 PMC2342859

[B16] Eckberg D. L. Eckberg M. J. (1982). Human sinus node responses to repetitive, ramped carotid baroreceptor stimuli. *Am. J. Physiol.* 242 H638–H644. 10.1152/ajpheart.1982.242.4.H638 7065276

[B17] Fox S. I. Rompolski K. (2019). *Human Physiology.* New York, NY: McGraw-Hill Education.

[B18] Fuller J. Wally C. Westermann-Long A. Korenfeld D. Carrell D. (2011). Resonance frequency measurements are reliable. *Appl. Psychophysiol. Biofeedback* 36:219.

[B19] Gevirtz R. (2013). The promise of heart rate variability biofeedback: evidence-based applications. *Biofeedback* 41 110–120. 10.5298/1081-5937-41.3.01

[B20] Gevirtz R. N. Lehrer P. M. Schwartz M. S. (2016). “Cardiorespiratory biofeedback,” in *Biofeedback: A Practitioner’s Guide*, eds Schwartz M. S. Andrasik F. (New York, NY: The Guilford Press), 196–213.

[B21] Giardino N. D. Lehrer P. M. Edelberg R. (2002). Comparison of finger plethysmograph to ECG in the measurement of heart rate variability. *Psychophysiology* 39 246–253. 10.1111/1469-8986.3920246 12212675

[B22] Goldberger A. L. (1991). Is the normal heartbeat chaotic or homeostatic? *News Physiol. Sci.* 6 87–91. 10.1152/physiologyonline.1991.6.2.87 11537649

[B23] Goldstein D. S. Bentho O. Park M. Y. Sharabi Y. (2011). Low frequency power of heart rate variability is not a measure of cardiac sympathetic tone but may be a measure of modulation of cardiac autonomic outflows by baroreflexes. *Exp. Physiol.* 96 1255–1261. 10.1113/expphysiol.2010.056259 21890520 PMC3224799

[B24] Grossman P. Taylor E. W. (2007). Toward understanding respiratory sinus arrhythmia: relations to cardiac vagal tone, evolution and biobehavioral functions. *Biol. Psychol.* 74 263–285. 10.1016/j.biopsy 17081672

[B25] Gulliksen H. (1987). *Theory of Mental Tests.* Hillsdale, NJ: Erlbaum.

[B26] Jan H. Y. Chen M. F. Fu T. C. Lin W. C. Tsai C. L. Lin K. P. (2019). Evaluation of coherence between ECG and PPG derived parameters on heart rate variability and respiration in healthy volunteers with/without controlled breathing. *J. Med. Biol. Eng.* 39 783–795. 10.1007/s40846-019-00468-9

[B27] Karemaker J. M. (2009). Counterpoint: respiratory sinus arrhythmia is due to the baroreflex mechanism. *J. Appl. Psychol.* 106 1742–1743. 10.1152/japplphysiol.91107.2008a 19414625

[B28] Khazan I. (2019a). *Biofeedback and Mindfulness in Everyday Life: Practical Solutions for Improving Your Health and Performance.* New York, NY: W. W. Norton & Company. 10.1007/s10484-022-09564-0 36269523

[B29] Khazan I. (2019b). “A guide to normal values in biofeedback,” in *Physiological Recording Technology and Applications in Biofeedback and Neurofeedback*, eds Moss D. Shaffer F. (Oakbrook Terrace, IL: Association for Applied Psychophysiology and Biofeedback), 2–6.

[B30] Khazan I. Z. (2013). *The Clinical Handbook of Biofeedback: A Step-By-Step Guide for Training and Practice with Mindfulness.* Malden, MA: Wiley-Blackwell 10.1002/9781118485309

[B31] Kleiger R. E. Stein P. K. Bigger J. T. Jr. (2005). Heart rate variability: measurement and clinical utility. *Ann. Noninvasive Electrocardiol.* 10 88–101. 10.1111/j.1542-474X.2005.10101.x 15649244 PMC6932537

[B32] Koenig J. Thayer J. F. (2016). Sex differences in healthy human heart rate variability: a meta-analysis. *Neurosci. Biobehav. Rev.* 64 288–310. 10.1016/j.neubiorev.2016.03.007 26964804

[B33] Koh J. Brown T. E. Beightol L. A. Eckberg D. L. (1998). Contributions of tidal lung inflation to human R-R interval and arterial pressure fluctuations. *J. Auton. Nerv. Syst.* 68 89–95. 10.1016/s0165-1838(97)00114-8 9531448

[B34] Kollai M. Mizsei G. (1990). Respiratory sinus arrhythmia is a limited measure of cardiac parasympathetic control in man. *J. Physiol.* 434 329–342. 10.1113/jphysiol.1990.sp018070 2391653 PMC1189816

[B35] Kougias P. Weakley S. M. Yao Q. Lin P. H. Chen C. (2010). Arterial baroreceptors in the management of systemic hypertension. *Med. Sci. Monit.* 16 RA1–RA8. 20037502 PMC2921195

[B36] Kromenacker B. W. Sanova A. A. Marcus F. I. Allen J. J. B. Lane R. D. (2018). Vagal mediation of low-frequency heart rate variability during slow yogic breathing. *Psychosom. Med.* 80 581–587. 10.1097/psy.0000000000000603 29771730

[B37] Laborde S. Mosley E. Mertgen A. (2018). Vagal tank theory: the three Rs of cardiac vagal control functioning – resting, reactivity, and recovery. *Front. Neursci.* 12:458. 10.3389/fnins.2018.00458 30042653 PMC6048243

[B38] Laborde S. Mosley E. Thayer J. F. (2017). Heart rate variability and cardiac vagal tone in psychophysiological research – recommendations for experiment planning, data analysis, and data reporting. *Front. Psychol.* 8:213. 10.3389/fpsyg.2017.00213 28265249 PMC5316555

[B39] Lehrer P. (2013). How does heart rate variability biofeedback work? resonance, the baroreflex, and other mechanisms. *Biofeedback* 41 26–31. 10.5298/1081-5937-41.1.02

[B40] Lehrer P. Eddie D. (2013). Dynamic processes in regulation and some implications for biofeedback and biobehavioral interventions. *Appl. Psychophysiol. Biofeedback* 38 143–155. 10.1007/s10484-013-9217-6 23572244 PMC3699855

[B41] Lehrer P. Kaur K. Sharma A. Shah K. Huseby R. Bhavsar J. (2020a). Heart rate variability biofeedback improves emotional and physical health and performance: a systematic review and meta-analysis. *Appl. Psychophysiol. Biofeedback* 45 109–129. 10.1007/s10484-020-09466-z 32385728

[B42] Lehrer P. Vaschillo B. Zucker T. Graves J. Katsamanis M. Aviles M. (2013). Protocol for heart rate variability biofeedback training. *Biofeedback* 41 98–109. 10.5298/1081-5937-41.3.08

[B43] Lehrer P. M. Gevirtz R. (2014). Heart rate variability: How and why does it work? *Front. Psychol.* 5:756. 10.3389/fpsyg.2014.00756 25101026 PMC4104929

[B44] Lehrer P. M. Vaschillo E. (2008). The future of heart rate variability biofeedback. *Biofeedback* 36 11–14.

[B45] Lehrer P. M. Vaschillo E. Vaschillo B. Lu S.-E. Eckberg D. L. Edelberg R. (2003). Heart rate variability biofeedback increases baroreflex gain and peak expiratory flow. *Psychosom. Med.* 65 796–805. 10.1097/01.PSY.0000089200.81962.19 14508023

[B46] Lehrer P. M. Vaschillo E. Vaschillo B. Lu S.-E. Scardella A. Siddique M. (2004). Biofeedback treatment for asthma. *Chest* 126 352–361. 10.1378/chest.126.2.352 15302717

[B47] Lehrer P. M. Vaschillo E. Vidali V. (2020b). Heart rate and breathing are not always in phase during resonance frequency breathing. *Appl. Psychophysiol. Biofeedback* 45 145–152. 10.1007/s10484-020-09459-y 32285231

[B48] Lin G. Xiang Q. Fu X. Wang S. Wang S. Chen S. (2012). Heart rate variability biofeedback decreases blood pressure in prehypertensive subjects by improving autonomic function and baroreflex. *J. Altern. Complement. Med.* 18 143–152. 10.1089/acm.2010.0607 22339103

[B49] Mather M. Thayer J. (2018). How heart rate variability affects emotion regulation brain networks. *Curr. Opin. Behav. Sci.* 19 98–104. 10.1016/j.cobeha.2017.12.017 29333483 PMC5761738

[B50] McCraty R. Shaffer F. (2015). Heart rate variability: new perspectives on physiological mechanisms, assessment of self-regulatory capacity, and health risk. *Glob. Adv. Health Med.* 4 46–61. 10.7453/gahmj.2014.073 25694852 PMC4311559

[B51] Meehan Z. Muesenfechter N. Gravett N. Watson T. Smith A. Shearman S. (2017). A 1:2 inhalation-to-exhalation ratio does not increase heart rate variability during 6-bpm breathing [Abstract]. *Appl. Psychophysiol. Biofeedback* 45 110–111. 10.1007/s10484-018-9390-8 29541903

[B52] Noble D. J. Hochman S. (2019). Hypothesis: pulmonary afferent activity patterns during slow, deep breathing contribute to the neural induction of physiological relaxation. *Front. Physiol.* 10:1176. 10.3389/fphys.2019.01176 31572221 PMC6753868

[B53] Nunan D. Sandercock G. R. H. Brodie D. A. (2010). A quantitative systematic review of normal values for short-term heart rate variability in healthy adults. *Pacing Clin. Electrophysiol.* 33 1407–1417. 10.1111/j.1540-8159.2010.02841.x 20663071

[B54] Peltola M. A. (2012). Role of editing of R–R intervals in the analysis of heart rate variability. *Front. Physiol.* 3:148. 10.3389/fphys.2012.00148 22654764 PMC3358711

[B55] Penttilä J. Helminen A. Jartti T. Kuusela T. Huikuri H. V. Tulppo M. P. (2001). Time domain, geometrical and frequency domain analysis of cardiac vagal outflow: effects of various respiratory patterns. *Clin. Physiol.* 21, 365–376. 10.1046/j.1365-2281.2001.00337.x 11380537

[B56] Peper E. Gibney K. H. Tylova H. Harvey R. Combatalade D. (2008). *Biofeedback Mastery: An Experiential Teaching and Self-Training Manual.* Wheat Ridge, CO: AAPB.

[B57] Pinsky M. R. (2018). Cardiopulmonary interactions: physiological basis and clinical applications. *Ann. Am. Thorac. Soc.* 15 S45–S48. 10.1513/AnnalsATS.201704-339FR 28820609 PMC5822394

[B58] Porges S. W. (1995). Orienting in a defensive world: mammalian modifications of our evolutionary heritage. A polyvagal theory. *Psychophysiology* 32 301–318. 10.1111/j.1469-8986.1995.tb01213.x 7652107

[B59] Reyes del Paso G. A. Langewitz W. Mulder L. J. M. Van Roon A. Duschek S. (2013). The utility of low frequency heart rate variability as an index of sympathetic cardiac tone: a review with emphasis on a reanalysis of previous studies. *Psychophysiology* 50 477–487. 10.1111/psyp.12027 23445494

[B60] Russo M. A. Santarelli D. M. O’Rourke D. (2017). The physiological effects of slow breathing in the healthy human. *Breathe* 13 298–309. 10.1183/20734735.009817 29209423 PMC5709795

[B61] Sakakibara M. Kaneda M. Oikawa L. O. (2020). Efficacy of paced breathing at the low-frequency peak on heart rate variability and baroreflex sensitivity. *Appl. Psychophysiol. Biofeedback* 45 31–37. 10.1007/s10484-019-09453-z 31781925

[B62] Schafer A. Vagedes J. (2013). How accurate is pulse rate variability as an estimate of heart rate variability? a review on studies comparing photoplethysmographic technology with an electrocardiogram. *Int. J. Cardiol.* 166 15–29. 10.1016/j.ijcard.2012.03.119 22809539

[B63] Segerstrom S. C. Ness L. S. (2007). Heart rate variability reflects self-regulatory strength, effort, and fatigue. *Psychol. Sci.* 18 275–281. 10.1111/j.1467-9280.2007.01888.x 17444926

[B64] Shaffer F. (2020). Resonance frequency assessment: the challenge of standardizing heart rate variability biofeedback research. *Biofeedback* 48 7–15. 10.5298/1081-5937-48.01.06

[B65] Shaffer F. Combatalade D. (2013). Don’t add or miss a beat: a guide to cleaner heart rate variability recordings. *Biofeedback* 41 121–130. 10.5298/1081-5937-41.3.04

[B66] Shaffer F. Ginsberg J. P. (2017). An overview of heart rate variability (HRV) metrics and norms. *Front. Public Health* 5:258. 10.3389/fpubh.2017.00258 29034226 PMC5624990

[B67] Shaffer F. McCraty R. Zerr C. L. (2014). A healthy heart is not a metronome: an integrative review of the heart’s anatomy and heart rate variability. *Front. Psychol.* 5:1040. 10.3389/fpsyg.2014.01040 25324790 PMC4179748

[B68] Shaffer F. Moss D. (2019). “Biofeedback,” in *Brain and Heart Dynamics*, eds Bugada D. Bellini V. Bignami E. G. Lorini L. F. (Cham: Springer), 1–15.

[B69] Shaffer F. Shearman S. Meehan Z. Gravett N. Urban H. (2019). “The promise of ultra-short-term (UST) heart rate variability measurements: a comparison of Pearson product-moment correlation coefficient and limits of agreement (LoA) concurrent validity criteria,” in *Physiological Recording Technology and Applications in Biofeedback and Neurofeedback*, eds Moss D. Shaffer F. (Oakbrook Terrace, IL: Association for Applied Psychophysiology and Biofeedback), 214–220.

[B70] Steffen P. R. Austin T. DeBarros A. Brown T. (2017). The impact of resonance frequency breathing on measures of heart rate variability, blood pressure, and mood. *Front. Public Health* 5:222. 10.3389/fpubh.2017.00222 28890890 PMC5575449

[B71] Stein P. K. Domitrovich P. P. Hui N. Rautaharju P. Gottfdiener J. (2005). Sometimes higher heart rate variability is not better heart rate variability: results of graphical and nonlinear analyses. *J. Cardiovasc. Electrophysiol.* 16 954–959. 10.1111/j.1540-8167.2005.40788.x 16174015

[B72] Strauss-Blasche G. Moser M. Voica M. McLeod D. Klammer N. Marktl W. (2000). Relative timing of inspiration and expiration affects respiratory sinus arrhythmia. *Clin. Exp. Pharmacol. Physiol.* 27 601–606. 10.1046/j.1440-1681.2000.03306.x 10901389

[B73] Swenne C. A. (2013). Baroreflex sensitivity: mechanisms and measurement. *Neth. Heart J.* 21 58–60. 10.1007/s12471-012-0346-y 23179611 PMC3547418

[B74] Taha B. H. Simon P. M. Dempsey J. A. Skatrud J. B. Iber C. (1995). Respiratory sinus arrhythmia in humans: an obligatory role for vagal feedback from the lungs. *J. Appl. Physiol.* 78 638–645. 10.1152/jappl.1995.78.2.638 7759434

[B75] Tan G. Shaffer F. Lyle R. Teo I. (eds) (2016). *Evidence-Based Practice in Biofeedback and Neurofeedback*, 3rd Edn Wheat Ridge, CO: Association for Applied Psychophysiology.

[B76] Task Force Report. (1996). Heart rate variability: standards of measurement, physiological interpretation, and clinical use. *Circulation* 93 1043–1065. 10.1161/01.CIR.93.5.1043 8598068

[B77] Thayer J. F. Lane R. D. (2000). A model of neurovisceral integration in emotion regulation and dysregulation. *J. Affect. Disord.* 61 201–216. 10.1016/S0165-0327(00)00338-4 11163422

[B78] Thayer J. F. Ǻhs F. Fredrikson M. Sollers J. J. III Wager T. D. (2012). A meta-analysis of heart rate variability and neuroimaging studies: implications for heart rate variability as a marker of stress and health. *Neurosci. Biobehav. Rev.* 36 747–756. 10.1016/j.neubiorev.2011.11.009 22178086

[B79] Thayer J. F. Hansen A. L. Saus-Rose E. Johnson B. H. (2009). Heart rate variability, prefrontal neural function, and cognitive performance: the neurovisceral integration perspective on self-regulation, adaptation, and health. *Ann. Behav. Med.* 37, 141–153. 10.1007/s12160-009-9101-z 19424767

[B80] Umetani K. Singer D. H. McCraty R. Atkinson M. (1998). Twenty-four hour time domain heart rate variability and heart rate: relations to age and gender over nine decades. *J. Am. Coll. Cardiol.* 31 593–601. 10.1016/S0735-1097(97)00554-8 9502641

[B81] Vaillancourt D. E. Newell K. M. (2002). Changing complexity in human behavior and physiology through aging and disease. *Neurobiol. Aging* 23 1–11. 10.1016/S0197-4580(01)00247-0 11755010

[B82] Van Diest I. Verstappen K. Aubert A. E. Widjaja D. Vansteenwegen D. Vlemincx E. (2014). Inhalation/exhalation ratio modulates the effect of slow breathing on heart rate variability and relaxation. *Appl. Psychophysiol. Biofeedback* 39 171–180. 10.1007/s10484-014-9253-x 25156003

[B83] Vaschillo E. Lehrer P. Rishe N. Konstantinov M. (2002). Heart rate variability biofeedback as a method for assessing baroreflex function: a preliminary study of resonance in the cardiovascular system. *Appl. Psychophysiol. Biofeedback* 27 1–27. 10.1023/A:1014587304314 12001882

[B84] Vaschillo E. G. Vaschillo B. Lehrer P. M. (2004). Heartbeat synchronizes with respiratory rhythm only under specific circumstances. *Chest* 126 1385–1386. 10.1016/S0012-3692(15)31329-5 15486413

[B85] Vaschillo E. G. Vaschillo B. Lehrer P. M. (2006). Characteristics of resonance in heart rate variability stimulated by biofeedback. *Appl. Psychophysiol. Biofeedback* 31 129–142. 10.1007/s10484-006-9009-3 16838124

[B86] Vaschillo E. G. Vaschillo B. Pandina R. J. Bates M. E. (2011). Resonances in the cardiovascular system caused by rhythmical muscle tension. *Psychophysiology* 48 927–936. 10.1111/j.1469-8986.2010.01156.x 21143610 PMC3094735

[B87] Weinschenk S. W. Beise R. D. Lorenz J. (2016). Heart rate variability (HRV) in deep breathing tests and 5-min short-term recordings: agreement of ear photoplethysmography with ECG measurements, in 343 subjects. *Eur. J. Appl. Physiol.* 116 1527–1535. 10.1007/s00421-016-3401-3 27278521

[B88] Yasuma F. Hayano J. (2004). Respiratory sinus arrhythmia: Why does the heartbeat synchronize with respiratory rhythm? *Chest* 125 638–690. 10.1378/chest.125.2.683 14769752

[B89] Zaccaro A. Piarulli A. Laurino M. Garbella E. Menicucci D. Neri B. (2018). How breath-control can change your life: a systematic review on psycho-physiological correlates of slow breathing. *Front. Hum. Neurosci.* 12:353. 10.3389/fnhum.2018.00353 30245619 PMC6137615

[B90] Zautra A. J. Fasman R. Davis M. C. Craig A. D. B. (2010). The effects of slow breathing on affective responses to pain stimuli: an experimental study. *Pain* 149 12–18. 10.1016/j.pain.2009.10.001 20079569

[B91] Zerr C. Kane A. Vodopest T. Allen J. Hannan J. Fabbri M. (2015). Does inhalation-to-exhalation ratio matter in heart rate variability biofeedback? [Abstract]. *Appl. Psychophysiol. Biofeedback* 40:135.

