# Corrigendum: A Practical Guide to Resonance Frequency Assessment for Heart Rate Variability Biofeedback

**DOI:** 10.3389/fnins.2020.627512

**Published:** 2020-12-01

**Authors:** Fred Shaffer, Zachary M. Meehan

**Affiliations:** ^1^Center for Applied Psychophysiology, Truman State University, Kirksville, MO, United States; ^2^Department of Psychological and Brain Sciences, University of Delaware, Newark, DE, United States

**Keywords:** biofeedback, complexity, emotional self-regulation, heart rate variability, neurocardiology, resonance, performance

In the original article, there was an error. The high-frequency band's upper limit was reversed.

A correction has been made to “A Resonance Frequency Assessment Protocol section,” “Terms and Definitions subsection.”

Data from heart rate, respiration, and their synchrony provide detailed information for resonance frequency assessment. Clinicians who assess resonance frequency are concerned with the smoothness and regularity of heart rate signals. Heart rate-respiration phase synchrony is the phase angle of the peaks and troughs of the heart rate and respiration rate signals. HRV frequency-domain metrics calculate the spectral distribution of signal energy. HRV biofeedback is concerned with the very-low-frequency (VLF; 0.0033–0.04 Hz), low-frequency (LF; 0.04–0.15 Hz), and high-frequency (HF; 0.15–0.40 Hz) bands. Although there is uncertainty regarding the sources of VLF power in short-term measurements (Kleiger et al., 2005), sympathetic activation due to effortful breathing is a possible source (Bernardi et al., 1996). There is also disagreement about the sources of LF power in short-term measurements (Akselrod et al., 1981; Goldstein et al., 2011; Reyes del Paso et al., 2013). LF power is an important indicator of HRV biofeedback training success for several reasons. First, slow paced breathing, which is one method of stimulating the baroreflex, increases LF power by increasing cardiac vagal tone (Kromenacker et al., 2018). Second, increased LF power is associated with greater RSA and HRV (Vaschillo et al., 2002). Increased RSA and HRV occur in the LF range because the baroreceptor reflex's resonance frequency resides within this range (Lehrer et al., 2020). HF power is due to parasympathetic activity, and the natural logarithm of HF power indexes cardiac vagal tone (Task Force Report, 1996). Absolute power is the signal energy within a frequency band expressed in ms^2^/Hz. Normalized power is the percentage of total power. For example, normalized LF power is LF/(LF + HF) or LF/(VLF + LF + HF). Peaks are the highest-amplitude frequencies within a band like LF. Resonance frequency assessment examines both the magnitude and number of LF peaks (Shaffer and Ginsberg, 2017). LF peak amplitude and the number of LF peaks are among six resonance frequency selection criteria (Lehrer et al., 2013). Larger peaks indicate greater resonance effects due to increased breathing and heart rate synchrony. Several LF peaks may occur when individuals breathe at a single rate outside of their resonance frequency. This can produce separate peaks at the baroreflex frequency and their actual respiratory rate. While breathing at adjacent rates may still stimulate the baroreflex, it will produce smaller resonance effects (Lehrer et al., 2020). When clients do not precisely follow instructions, changing respiration rates can also produce multiple peaks and weaker resonance effects. Breathing in a narrow frequency range around the resonance frequency better stimulates the baroreflex and increases RSA than breathing in a wider frequency range (Vaschillo et al., 2002).

The authors apologize for this error and state that this does not change the scientific conclusions of the article in any way. The original article has been updated.
